# Vitamin D Status and Chronic Obstructive Pulmonary Disease: A Prospective General Population Study

**DOI:** 10.1371/journal.pone.0090654

**Published:** 2014-03-04

**Authors:** Tea Skaaby, Lise Lotte Nystrup Husemoen, Betina Heinsbæk Thuesen, Charlotta Pisinger, Torben Jørgensen, Runa Vavia Fenger, Allan Linneberg

**Affiliations:** 1 Research Centre for Prevention and Health, Glostrup University Hospital, Glostrup, Denmark; 2 Faculty of Health Science, University of Copenhagen, Copenhagen, Denmark; 3 Faculty of Medicine, Aalborg University, Aalborg, Denmark; 4 Department of Clinical Experimental Research, Glostrup University Hospital, Glostrup, Denmark; 5 Department of Clinical Medicine, Faculty of Health and Medical Sciences, University of Copenhagen, Copenhagen, Denmark; New York University, United States of America

## Abstract

**Objectives:**

Vitamin D deficiency is common among persons with chronic obstructive pulmonary disease (COPD). Whether vitamin D affects the development and deterioration of COPD or is a consequence of the disease lacks clarity. We investigated the association between vitamin D status and prevalent and incident COPD in the general population.

**Methods:**

We included a total of 12,041 individuals from three general population studies conducted in 1993–94, 1999–2001, and 2006–2008, respectively, with vitamin D measurements. Information on COPD was obtained from the Danish National Patient Register and The Danish Registry of Causes of Death.

**Results:**

There were 85 prevalent and 463 incident cases of COPD (median follow-up 9.7 years). We found a statistically significant inverse association between vitamin D status and prevalent COPD with odds ratio = 0.89 (95% confidence interval, CI: 0.79, 1.0), but no statistically significant association with incident COPD with a hazard ratio = 0.98 (95% CI: 0.94, 1.0), respectively, per 10 nmol/l higher vitamin D status, when adjusted for possible confounders.

**Conclusions:**

We found a statistically significant inverse cross-sectional association between vitamin D status and COPD, but no association between vitamin D status and incident COPD.

## Introduction

Chronic obstructive pulmonary disease (COPD) is an important cause of mortality and morbidity and the fifth leading cause of death in the world [1]. It is characterized by an irreversible air flow loss thought to be due to an inflammatory airway destruction brought about by airway irritants and noxious gases such as the components of tobacco smoke [2]. The most frequent symptoms are sputum production, shortness of breath and a productive cough. Acute exacerbations –a sudden worsening of symptoms— are also manifestations of the disease. Most exacerbations are believed to be triggered by viral and bacterial respiratory infections [3], and they are associated with an increased mortality risk [4]. The diagnosis of COPD is based on post-bronchodilator lung function measurement. Spirometry measures the forced expiratory volume in one second (FEV1) and the forced vital capacity (FVC). According to the ERS criteria, FEV1% predicted <88% for men, or <89% for women is diagnostic, whereas according to the 2011 GOLD criteria, a FEV1/FVC ratio of less than 70% defines a person as having COPD [5]; [6].

Vitamin D is produced in sun-exposed skin and to a smaller extent derived from the diet and dietary supplements. It has a well-established role in preserving skeletal integrity. Recent studies, however, have emphasized the role of vitamin D and the vitamin D receptor in the regulation of genes involved in immunity, inflammation and cellular proliferation. Vitamin D is thought to affect immunity through several pathways. To name a few, it affects a great number of genes in leucocytes and increases the synthesis of antimicrobial peptides [7], regulates the adaptive lymphocyte responses, and reduces the production of inflammatory cytokines which surprisingly results in a strengthened antimicrobial response [8].

Observational studies have found a high prevalence of vitamin D deficiency among COPD patients [9], and pulmonary function shows correlation with low vitamin D status as does incidence of pulmonary infections [10]; [11]. Vitamin D could affect the pathogenesis and severity of COPD in a number of ways. By means of its role in immunity, vitamin D may affect the frequency of respiratory infections triggering the acute exacerbations in COPD. In addition, it may lead to more severe exacerbations due to an impaired response to the pathogen causing an excessive adaptive immune response with a higher synthesis of cytokines and increased bronchial inflammation [8]. Further, it inhibits the proliferation of airway smooth muscle [8] and possibly influences tissue remodeling through collagen synthesis [12],

We hypothesized that low vitamin D status could be associated with a higher risk of having and developing COPD. We investigated the cross-sectional as well as the prospective associations between vitamin D status as assessed by serum 25-hydroxyvitamin D (25-OH-D) and fatal and non-fatal COPD according to The International Classification of disease in three random samples of the general population.

## Materials and Methods

### Ethics Statement

Participants gave their informed written consent, and the studies were approved by the Ethics Committee of Copenhagen and the Danish Data Protection Agency. The recommendations of the Declaration of Helsinki were followed.

### Study populations

We used the three population based studies, Monica10, Inter99, and Health2006, recruited from the Danish Central Personal Register as random samples of the population in the southern part of the former Copenhagen County at different time points. The studies included physical examinations, blood tests and questionnaires. The Monica10 study is a 10-year follow-up study of the Monica1 study conducted in 1982–1984 and including examinations of 3,785 individuals of Danish origin. The follow-up study (1993–94) included 2,656 individuals between 40–71 years yielding a participation rate of 64.3% [13].

The Inter99 study conducted in 1999–2001 and including 6,784 individuals aged 30–60 years from a general population had a baseline participation rate of 52.5% [14]. The Inter99 study is a population-based randomized controlled trial (CT00289237, ClinicalTrials.gov) investigating the effects of lifestyle intervention on cardiovascular disease [14].

In the Health2006 study, a sample of 7,931 Danish citizens aged 18 to 69 years, was invited to a general health examination [15]. A total of 3,471 (43.8%) persons were examined between June 2006 and June 2008. There was an overlap of 163 participants in the Inter99 and the Health2006 studies. These participants were excluded from the analyses of the Health2006 study. In total, we included 2,649, 6,146, and 3,246 participants from the Monica10, the Inter99, and the Health2006 study, respectively, with measurements of vitamin D status.

### Vitamin D analysis

Serum samples from the participants in the Monica10 and the Inter99 studies were stored at −20°C, while the samples from the Health2006 study were stored at −80°C until the analyses of 25-OH-D in 2009, 2010, and 2011, respectively. In the Monica10 study, measurements of serum 25-OH-D were performed with the IDS-SYS 25-Hydroxy Vitamin D method with the IDS-iSYS Multi-Discipline System (IDS Nordic A/S, Herlev, Denmark) [16]. Measurements of 25-OH-D in the Inter99 study were performed by high performance liquid chromatography [17]. In the Health2006 study, 25-OH-D was measured by immunoassay using Cobas e411 (Roche Diagnostics, Mannheim, Germany) [18].

### Outcome

Residents in Denmark have a personal civil registration number which is permanent and unique and allows linkage of data from complete national registers on an individual level. Information on diseases according to the *International Classification of Diseases* (ICD) was obtained from the Danish National Patient Register [19]. The register holds information on every admission to Danish hospitals since 1978. Each admission is registered by a primary diagnosis and optionally one or more secondary diagnoses. The Danish Registry of Causes of Death provided up to three diagnoses presumed to be the cause of death [20]. The included diagnoses were the ICD10 and ICD8 diagnoses of COPD (ICD10: J42-J44 and ICD8: 491-492). Fatal and non-fatal COPD were combined. Participants with a registry-based diagnosis of COPD before baseline were excluded from the prospective studies. Participants were followed until 31 December 2010. Information on emigration status was obtained from the Danish Civil Registration System.

### Covariates

The questionnaires provided information on education, dietary habits, leisure time physical activity, smoking habits, alcohol consumption and intake of fish. Height and weight were measured without shoes and with light clothes. Body mass index (BMI) was calculated as weight (kg) divided by height (m) squared.

In the Monica10 study, spirometry was performed by the Vitalograph (Vitalograph, Buckingham, UK). In the Inter99 study, spirometry was performed by using the Cardiosoft software (GE Medical Systems, Freiburg, Germany) and a LF501 respiration flow transducer (Erich Jaeger B.V. and Marquette Hellige GmbH, Freiburg, Germany). In the Health2006 study, spirometry was performed with the SpiroUSB (MicroMedical Ltd, Rochester, UK) [21]. In all 3 studies, measurements of forced expiratory volume in one second (FEV1) were recorded, and the measured FEV1 in percentage of the predicted value was calculated [22].

The following categorizations of covariates were used: age (≤45, 45–55, or ≥55 years), study cohort (Monica10, Inter99, or Health2006); season of blood sample (March-May, June-August, September-November, or December-February); education/vocational training assessed by the question: “Do you have vocational training?” (no education, education including students); intake of fish (< twice a week, ≥ twice a week); physical activity during leisure time (sedentary, light, or moderate/vigorous); smoking habits (never smokers, ex-smokers, occasional smokers; current smokers <15 g/day; 15–<25 g/day, or ≥25 g of tobacco/day; 1 cigarette  = 1 g, 1 cheroot  = 2 g, 1 cigar  = 3 g, pipe = stated in g); alcohol consumption (0, >0–7, >7–14, or >14 drinks per week); BMI (<18.5 kg/m^2^, ≥18.5–25 kg/m^2^, ≥25–30 kg/m^2^, and ≥30 kg/m^2^), and FEV1 % predicted (<60%, 60–80%, >80%).

### Statistical analyses

All analyses were performed with SAS, version 9.2 (SAS Institute Inc. Cary, NC USA). P-values were two-sided, and p-values <0.05 were considered statistically significant. Descriptive characteristics and vitamin D status in the three study populations are presented as median (interquartile range, IQR) and compared with the non-parametric Kruskal Wallis test (table 1). Covariates according to COPD diagnosis at any time presented as % (number) were compared with the chi-squared test (table 2).

**Table 1:** Baseline characteristics and vitamin D status in the three study populations.

	Monica10		Inter99		Health2006	
Characteristics	N	25-OH-D, nmol/l median (IQR)	N	25-OH-D, nmol/l median (IQR)	N	25-OH-D, nmol/l median (IQR)
Male gender	1329	61.9 (45.6, 81.7)	3006	47.0 (32.0, 64.0)	1470	40.7 (29.0, 53.7)
Female gender	1320	60.3 (43.8, 80.0)	3140	49.0 (33.0, 66.9)	1776	42.2 (29.7, 57.2)
		P = 0.287		P = 0.005		P = 0.011
≤45 years of age	726	62.2 (45.1, 82.1)	2780	49.0 (34.0, 65.0)	1244	39.7 (26.7, 54.4)
45–55 years of age	740	62.2 (44.9, 85.5)	2422	48.0 (32.0, 65.0)	759	40.4 (30.0, 53.2)
≥55 years of age	1183	58.9 (44.5, 77.1)	944	47.0 (31.0, 66.0)	1225	44.4 (31.4, 58.2)
		P = 0.025		P = 0.249		P<0.0001
Blood test Mar-May	419	50.0 (38.0, 65.6)	1814	43.0 (28.0, 59.0)	779	36.4 (25.2, 52.2)
Blood test Jun-Aug	611	76.5 (55.4, 96.6)	1387	53.0 (35.0, 77.0)	716	48.0 (36.8, 62.2)
Blood test Sep-Nov	1150	64.1 (47.9, 82.1)	1601	57.0 (43.0, 71.0)	998	44.8 (33.2, 57.9)
Blood test Dec-Feb	469	50.1 (37.4, 65.2)	1344	42.0 (29.0, 54.0)	753	34.9 (22.5, 48.2)
		P<0.0001		P<0.0001		P<0.0001
Only basic education	677	56.3 (41.4, 74.8)	918	47.0 (32.0, 64.0)	439	42.9 (29.5, 58.4)
Education beyond basic	1971	62.8 (46.6, 83.1)	5033	48.0 (33.0, 65.0)	2757	41.2 (29.5, 55.4)
		P<0.0001		P = 0.258		P = 0.085
BMI <18.5 kg/m^2^	26	52.9 (43.2, 80.1)	66	46.5 (33.0, 76.0)	56	43.9 (20.5, 59.0)
BMI 18.5–24.9 kg/m^2^	1202	62.9 (46.1, 85.4)	2621	50.0 (34.0, 67.0)	1509	43.7 (31.4, 58.2)
BMI 25–29.9 kg/m^2^	1008	62.2 (46.5, 79.8)	2396	48.0 (33.0, 65.0)	1169	41.2 (29.0, 55.2)
BMI ≥30 kg/m^2^	413	52.8 (39.5, 71.0)	1059	43.0 (30.0, 59.0)	510	35.8 (24.7, 48.4)
		P<0.0001		P<0.0001		P<0.0001
Sedentary physical activity	550	54.6 (38.0, 76.6)	1243	45.0 (30.0, 63.0)	589	36.4 (24.7, 49.4)
Light physical activity	1478	60.2 (44.2, 79.4)	3779	48.0 (33.0, 65.0)	1943	41.7 (29.5, 55.4)
Moderate/vigorous physical activity	573	69.1 (52.9, 88.5)	1016	51.0 (36.0, 68.0)	677	45.4 (33.2, 59.7)
		P<0.0001		P<0.0001		P<0.0001
Intake of fish <twice a week	2119	61.0 (44.4, 81.3)	3454	48.0 (32.0, 65.0)	661	41.9 (28.5, 55.4)
Intake of fish ≥twice a week	344	64.6 (50.7, 83.6)	2670	48.0 (33.0, 65.0)	2543	41.2 (29.7, 55.9)
		P = 0.009		P = 0.352		P = 0.787
Never smoker	693	62.9 (46.1, 81.8)	2147	50.0 (34.0, 66.0)	1347	40.4 (27.7, 54.2)
Former smoker	727	65.1 (48.9, 81.8)	1572	50.0 (35.0, 68.0)	1039	40.4 (30.0, 54.2)
Occasional smoker	24	55.4 (36.5, 76.6)	218	47.0 (32.0, 64.0)	99	44.7 (31.2, 61.9)
Current smoker, <15 g/day	494	59.2 (43.7, 80.8)	633	48.0 (31.0, 65.0)	297	46.9 (33.7, 60.9)
Current smoker, <25 g/day	553	57.1 (41.1, 81.0)	1116	45.0 (29.0, 61.0)	326	44.8 (31.7, 60.2)
Current smoker, ≥25 g/day	150	52.9 (37.2, 72.1)	419	44.0 (28.0, 62.0)	105	41.2 (29.0, 59.7)
		P<0.0001		P<0.0001		P<0.0001
0 drinks of alcohol/week	358	55.7 (39.3, 74.7)	546	46.0 (29.0, 65.0)	192	36.2 (25.5, 49.2)
≤7 drinks of alcohol/week	1099	59.7 (44.5, 79.5)	2661	49.0 (34.0, 65.0)	1424	40.7 (28.2, 53.9)
≤14 drinks of alcohol/week	572	67.7 (49.3, 86.2)	1298	49.0 (35.0, 65.0)	665	44.7 (32.2, 58.9)
>14 drinks of alcohol/week	599	60.6 (44.6, 83.1)	1430	47.0 (31.0, 64.0)	670	45.2 (32.7, 58.9)
		P<0.0001		P = 0.024		P<0.0001
FEV1% predicted <60%	104	48.9 (33.0, 64.7)	66	46.5 (34.0, 65.0)	63	37.2 (25.7, 51.2)
FEV1% predicted 60–80%	230	54.5 (39.6, 72.8)	532	43.0 (28.0, 61.5)	269	40.4 (28.5, 57.2)
FEV1% predicted >80%	2309	62.3 (46.0, 81.9)	5122	49.0 (34.0, 66.0)	2880	41.4 (29.7, 55.7)
		P = <0.0001		P = <0.0001		P = 0.614

P-values are according to the Kruskal Wallis test.

Abbreviations: FEV1% predicted, forced expiratory volume in one second in % of predicted; IQR, interquartile range; 25-OH-D; 25-hydroxyvitamin D; BMI, body mass index.

**Table 2:** Covariates according to COPD diagnosis at any time.

	% (n)	% (n)	P-value
Characteristics	No diagnosis of COPD (n = 11493)	Diagnosis of COPD (n = 548)	Chi-square test
Study			
Monica10	90.2 (2390)	9.8 (259)	<0.0001
Inter99	96.6 (5935)	3.4 (211)	
Health2006	97.6 (3168)	2.4 (78)	
Gender			
Male	95.4 (5536)	4.6 (269)	0.674
Female	95.5 (5957)	4.5 (279)	
Age, years			
≤45	98.4 (4674)	1.6 (76)	<0.0001
45–55	95.7 (3753)	4.3 (168)	
≥55	91.0 (3049)	9.0 (303)	
Season of blood collection			
Mar-May	95.9 (2889)	4.1 (123)	0.190
Jun-Aug	94.8 (2573)	5.2 (141)	
Sep-Nov	95.3 (3574)	4.7 (175)	
Dec-Feb	95.8 (2457)	4.2 (109)	
Education			
No	91.5 (1862)	8.5 (172)	<0.0001
Yes	96.3 (9403)	3.7 (358)	
Body mass index, kg/m^2^			
<18.5	87.2 (129)	12.8 (19)	<0.0001
18.5–24.9	95.5 (5091)	4.5 (241)	
25–29.9	96.0 (4388)	4.0 (185)	
≥30	94.9 (1880)	5.1 (102)	
Physical activity			
Sedentary	93.1 (2217)	6.9 (165)	<0.0001
Light	95.9 (6908)	4.1 (292)	
Moderate/vigorous	97.2 (2203)	2.8 (63)	
Weekly intake of fish			
<twice	94.6 (5894)	5.4 (340)	<0.0001
≥twice	96.9 (5382)	3.1 (175)	
Smoking habits, g/day			
Never smoker	99.2 (4154)	0.8 (33)	<0.0001
Former smoker	96.6 (3223)	3.4 (115)	
Occasional smoker	98.2 (335)	1.8 (6)	
Current smoker, <15	92.8 (1321)	7.2 (103)	
Current smoker, <25	90.0 (1796)	10.0 (199)	
Current smoker, ≥25	87.4 (589)	12.6 (85)	
Alcohol, drinks/week			
0	93.5 (1025)	6.5 (71)	<0.0001
≤7	96.3 (4995)	3.7 (189)	
≤14	96.3 (2441)	3.7 (94)	
>14	94.2 (2542)	5.8 (157)	
FEV1% predicted			
<60%	42.9 (100)	57.1 (133)	<0.0001
60–80%	82.0 (845)	18.0 (186)	
>80%	98.0 (10101)	2.0 (210)	

Abbreviations: COPD, chronic obstructive pulmonary disease; FEV1% predicted, forced expiratory volume in one second in % of predicted.

Logistic regression analysis was used to model the cross-sectional association between vitamin D status and baseline COPD in three models (table 3). Model 1 is adjusted for study group. Model 2 is further adjusted for gender, age, season, and education. Model 3 is further adjusted for alcohol consumption, smoking habits, leisure time physical activity, intake of fish, and BMI.

**Table 3:** Multivariable logistic regression analyses of the cross-sectional association between vitamin D status and COPD (Number of events = 67, total number = 10,987).

Prevalent COPD	Model 1$ OR (95% CI)	Model 2& OR (95% CI)	Model 3¤ OR (95% CI)
per 10 nmol/l higher 25-OH-D	0.87 (0.78, 0.98)	0.87 (0.78, 0.98)	0.89 (0.79, 1.0)
	P = 0.017	P = 0.020	P = 0.043
1^st^ vitamin D quartile	1 (reference)	1 (reference)	1 (reference)
2^nd^ vitamin D quartile	0.44 (0.23, 0.85)	0.44 (0.23, 0.85)	0.44 (0.23, 0.88)
3^rd^ vitamin D quartile	0.37 (0.19, 0.75)	0.37 (0.18, 0.74)	0.41 (0.20, 0.86)
4^th^ vitamin D quartile	0.50 (0.27, 0.95)	0.51 (0.27, 0.96)	0.53 (0.27, 1.05)
	P_trend_ = 0.017	P_trend_ = 0.019	P_trend_ = 0.049

$ Adjusted for study population.

& Further djusted for gender, age, education, and season.

¤ Further adjusted for alcohol consumption, smoking, leisure time physical activity, intake of fish, and BMI.

Abbreviations: CI, confidence interval; COPD, chronic obstructive pulmonary disease; BMI, body mass index; OR, odds ratio; 25-OH-D; 25-hydroxyvitamin D.

Multivariable Cox regression analysis was used to determine the association of baseline vitamin D status and incident COPD and mortality. Estimates are presented as hazard ratios, HRs (95% confidence intervals, CIs). We used delayed entry and age as underlying time axis; age was included as a continuous variable here. Participants were followed and contributed with risk time until date of first diagnosis of COPD, death, date of emigration or 31 December 2010, whichever came first. Median follow-up time was 9.7 years: 14.7, 10.7, and 3.5 years for the Monica10, Inter99, and Health2006 study, respectively. Individuals with a diagnosis of COPD before baseline (n = 86) were excluded from the Cox regression analyses. Model 1 (table 4) is adjusted for study group. Model 2 is further adjusted for gender, season, and education. Model 3 is further adjusted for alcohol consumption, smoking, leisure time physical activity, intake of fish, and BMI. Model 4 is further adjusted for baseline FEV1 % predicted (continuous variable).

**Table 4:** Hazard ratios and 95% confidence intervals for the prospective associations between serum 25-OH vitamin D status and incident fatal and non-fatal COPD (person years at risk = 101,719, number of events = 375, total number = 10,523).

Fatal and non-fatal COPD¤	Model 1$ HR (95% CI)	Model 2& HR (95% CI)	Model 3% HR (95% CI)	Model 4€ HR (95% CI)
per 10 nmol/l higher 25-OH-D	0.91 (0.87, 0.95)	0.91 (0.87, 0.95)	0.94 (0.90,0.98)	0.98 (0.94, 1.0)
	P<0.0001	P<0.0001	P = 0.008	P = 0.376
1st vitamin D quartile	1 (reference)	1 (reference)	1 (reference)	1 (reference)
2nd vitamin D quartile	0.57 (0.43, 0.75)	0.57 (0.43, 0.75)	0.64 (0.48, 0.85)	0.73 (0.55, 0.97)
3rd vitamin D quartile	0.65 (0.49, 0.84)	0.64 (0.49, 0.84)	0.83 (0.63, 1.1)	1.0 (0.77, 1.4)
4th vitamin D quartile	0.49 (0.37, 0.66)	0.48 (0.36, 0.65)	0.61 (0.44, 0.83)	0.75 (0.55, 1.0)
	P_trend_<0.0001	P_trend_<0.0001	P_trend_ = 0.009	P_trend_ = 0.285

$ Adjusted for study population.

& Further adjusted for gender, education, and season.

% Further adjusted for alcohol consumption, smoking, leisure time physical activity, intake of fish, and BMI.

€ Further adjusted for baseline FEV1% predicted.

¤ Persons with a diagnosis of COPD before baseline were excluded. Complete case analysis.

Abbreviations: CI, confidence interval; COPD, chronic obstructive pulmonary disease; BMI, body mass index; HR, hazard ratio; 25-OH-D, 25-hydroxyvitamin D; FEV1% predicted, forced expiratory volume in one second in % of predicted.

Vitamin D status was used both as a continuous and a categorical variable (quartiles); vitamin D status was divided into quartiles before merging the studies. We tested for a possible non-linear relationship by adding vitamin D squared or divided into quartiles. There was no interaction between vitamin D status and study population.

## Results

In the crude baseline analyses shown in Table 1, vitamin D status was highest in the Monica10 study, followed by the Inter99 study and lowest in the Health2006 study. Vitamin D status was highest in summer and autumn and lowest in winter and spring, and vitamin D status was positively associated with physical activity in the three cohorts. Among current smokers, there seemed to be an inverse association between amount of smoking and vitamin D status. Persons with FEV1 % predicted >80% had higher vitamin D status than the rest.

There were 85 cases of COPD at baseline and a total of 463 incident cases of COPD during follow-up. Of the 463 incident cases of COPD, 13 were diagnoses of death and 450 were hospital diagnoses. Chronic bronchitis (J42) accounted for 4.5% (n = 21), emphysema (J43) for 3.0% (n = 14), and other chronic obstructive pulmonary disease (J44) for 92.5% (n = 428) of the incident diagnoses of COPD. Included in the group “other chronic obstructive pulmonary disease (J44)” were: chronic obstructive pulmonary disease with acute lower respiratory infection (J44.0); chronic obstructive pulmonary disease with acute exacerbation, unspecified (J44.1); other specified chronic obstructive pulmonary disease (J44.8); and chronic obstructive pulmonary disease, unspecified (J44.9).

Table 2 shows the distribution of selected covariates according to a COPD diagnosis at any time (i.e. both prevalent and incident COPD). A diagnosis of COPD was most frequent in the Monica10 study, followed by the Inter99 study, and least frequent in the Health2006 study. Increasing age, no education, BMI below normal, decreasing physical activity, a low intake of fish, and both a high intake of and alcohol abstinence were associated with a higher risk of having a diagnosis of COPD. Regarding smoking habits, never smokers had the lowest risk of COPD, followed by occasional smokers and former smokers. For current smokers, there seemed to be a dose-response relationship between amount of smoking and risk of COPD.

Logistic regression analyses of the cross-sectional relationship between baseline vitamin D status and prevalent COPD showed statistically significant lower odds ratios with higher vitamin D status (table 3), both when using vitamin D as a continuous variable and in quartiles and in both unadjusted and adjusted models. The estimates remained essentially unchanged after multivariable adjustments (table 3, model 3).

A higher vitamin D status was significantly associated with a lower hazard ratio of incident non-fatal and fatal COPD, both when using vitamin D as a continuous variable and in quartiles in both adjusted and unadjusted models, although the multivariable adjustments somewhat attenuated the estimates (model 1–3, table 4). However, after adjusting for the baseline value of FEV1 % predicted the estimate approached 1, and the association was no longer statistically significant (model 4, table 4).

Table 5 displays the analyses of the association between vitamin D status and incident COPD stratified by the baseline value of FEV1 % predicted. For the persons with FEV1 % predicted >80%, which is by far the largest group, the inverse association between vitamin D status and incident COPD is statistically significant in the unadjusted model and when adjusted for gender education and season (model 1–2, table 5), but does not reach statistical significance in the fully adjusted model (model 3, table 5). For the persons in the groups with FEV1 % predicted between 60-80% and <60% there is a HR below 1 of incident COPD with higher vitamin D status, never reaching statistical significance.

**Table 5:** Hazard ratios and 95% confidence intervals for the prospective associations between serum 25-OH vitamin D status and incident fatal and non-fatal COPD stratified by FEV1% predicted.

Fatal and non-fatal COPD¤	Number of events (total number)	Model 1$ HR (95% CI)	Model 2& HR (95% CI)	Model 3% HR (95% CI)
**FEV1% predicted<60%**	68 (145)			
per 10 nmol/l higher 25-OH-D		0.96 (0.87, 1.1)	0.95 (0.86, 1.1)	0.96 (0.85, 1.1)
		P = 0.408	P = 0.344	P = 0.501
1st vitamin D quartile		1 (reference)	1 (reference)	1 (reference)
2nd vitamin D quartile		0.95 (0.52, 1.7)	0.97 (0.52, 1.8)	1.4 (0.66, 2.9)
3rd vitamin D quartile		1.1 (0.55, 2.2)	0.94 (0.46, 1.9)	1.0 (0.49, 2.2)
4th vitamin D quartile		0.62 (0.29, 1.3)	0.59 (0.26, 1.3)	0.59 (0.24, 1.4)
		P_trend_ = 0.349	P_trend_ = 0.235	P_trend_ = 0.277
**FEV1% predicted 60–80%**	136 (885)			
per 10 nmol/l higher 25-OH-D		0.97 (0.90, 1.0)	0.97 (0.90, 1.0)	0.98 (0.91, 1.1)
		P = 0.367	P = 0.442	P = 0.650
1st vitamin D quartile		1 (reference)	1 (reference)	1 (reference)
2nd vitamin D quartile		0.58 (0.36, 0.93)	0.59 (0.37, 0.96)	0.56 (0.34, 0.92)
3rd vitamin D quartile		0.67 (0.42, 1.1)	0.69 (0.43, 1.1)	0.74 (0.45, 1.2)
4th vitamin D quartile		0.74 (0.47, 1.2)	0.75 (0.47, 1.2)	0.78 (0.47, 1.3)
		P_trend_ = 0.176	P_trend_ = 0.221	P_trend_ = 0.392
**FEV1% predicted >80%**	171 (9493)			
per 10 nmol/l higher 25-OH-D		0.92 (0.87, 0.98)	0.92 (0.86, 0.98)	0.96 (0.90, 1.0)
		P = 0.011	P = 0.013	P = 0.222
1st vitamin D quartile		1 (reference)	1 (reference)	1 (reference)
2nd vitamin D quartile		0.55 (0.35, 0.84)	0.55 (0.35, 0.84)	0.63 (0.41, 0.98)
3rd vitamin D quartile		0.79 (0.54, 1.2)	0.81 (0.55, 1.2)	1.0 (0.70, 1.6)
4th vitamin D quartile		0.51 (0.33, 0.79)	0.52 (0.33, 0.81)	0.68 (0.43, 1.1)
		P_trend_ = 0.018	P_trend_ = 0.025	P_trend_ = 0.405

¤ Persons with a diagnosis of COPD before baseline were excluded. Complete case analysis.

$ Adjusted for study population.

& Further adjusted for gender, education, and season.

% Further adjusted for alcohol consumption, smoking, leisure time physical activity, intake of fish, and BMI.

Abbreviations: CI, confidence interval; COPD, chronic obstructive pulmonary disease; BMI, body mass index; HR, hazard ratio; 25-OH-D, 25-hydroxyvitamin D; FEV1% predicted, forced expiratory volume in one second in % of predicted.

There were no sign of deviation from linearity in neither the cross-sectional nor the prospective regression analyses as tested by including a quadratic vitamin D term.

## Discussion

To the best of our knowledge, no previous study has investigated the association between vitamin D status and incident fatal and non-fatal COPD in the general population. In the cross-sectional analyses, we found statistically lower odds ratios of prevalent COPD with higher baseline vitamin D status. In the prospective analyses, we found a higher vitamin D status to be significantly associated with a lower hazard ratio of incident non-fatal and fatal COPD in the partly adjusted analyses, but the fully adjusted analyses yielded non-significantly lower risk of COPD, the estimates approaching 1. Likewise, the fully adjusted analyses stratified by FEV1 % predicted showed non-significantly lower risk of incident COPD.

There are several studies in favour of a role of vitamin D in pulmonary disease: We previously reported an inverse association between vitamin D status and death caused by respiratory disease [23]. Lange et al found vitamin D deficiency to be associated with lower lung function and faster lung function decline in smokers in a cohort of elderly men. Likewise, Persson et al found that COPD was associated with an increased risk of vitamin D deficiency in a case-control study [9]. Romme et al found that vitamin D deficiency was present in the majority of COPD patients in pulmonary rehabilitation [24], and Black et al found a dose-response relationship between the vitamin D status and FEV_1_ [10]. Also, a study by Ginde et al found an inverse association between vitamin status and recent upper respiratory tract infections [11].

However, against a role of vitamin D in the development and deterioration of COPD are the results from the following studies: Kunisaki et al found no association between vitamin D status and risk of acute exacerbations in a cohort of 973 COPD patients [25]. A prospective cohort study by Holmgaard et al reported that vitamin D status in 462 patients with COPD was not associated with mortality [26]. Likewise, a RCT of 182 patients with moderate to very severe COPD showed that high-dose supplementation did not reduce the incidence of exacerbations, although it may reduce exacerbations in participants with severe vitamin D deficiency [27].

The observed association between vitamin D status and COPD may be due to reverse causation (i.e. that COPD patients develop a low vitamin D status as a consequence of the disease rather that vitamin D status affecting the development and progression of the disease), as persons with COPD are more prone to vitamin D deficiency for several reasons: the physical impairment leads to reduced outdoor activity; old age which is associated with low vitamin status; glucocorticoid treatment inducing vitamin D catabolism; and malnutrition [9]. Also, COPD patients have accelerated skin ageing caused by smoking, renal impairment, and reduced fat tissue due to wasting, which may decrease the amount of vitamin D produced in the skin [9].

The strengths of our study include the registry-based diagnoses of fatal and non-fatal COPD with a long-term follow-up with almost no individuals lost to follow-up; the population-based design which is thought to be less prone to bias compared with patient-based designs; the wide distribution of vitamin D status in the population [17]; and the available information on potential confounders. A particular strength is the fact that we adjusted for baseline FEV1% predicted although the FEV_1_/FVC ratio may also be important.

The limitations of the study include the single vitamin D measurement, which may lose predictive power over time [28]–[30], and the risk of residual confounding characteristic for observational studies. Since we only had a total of 67 cases in the cross-sectional analysis, it may be speculated whether the estimate is in fact fully adjusted by the covariates (Table 3). Having no information on vitamin D supplements would most likely attenuate any true association. The observation that a diagnosis of COPD was most frequent in the Monica10 study and least frequent in the Health2006 study is not surprising since the participants in Monica10 were followed for much longer than the Health2006 (and Inter99) participants. Also, the Monica10 participants were older.

The Danish National Patient Register is considered the best of its kind internationally and is overall a sound data source [31]. Regarding diagnoses of COPD in particular, Thomsen et al examined the validity and underreporting in the Danish National Patient Registry and [32]. They found that the positive predictive value for COPD was 92%, based on medical history, clinical symptoms and findings and spirometry results. However, they also found a substantial underreporting of COPD among patients admitted with other acute respiratory disorders like respiratory failure and pneumonia of whom 19% turned out to have COPD. Therefore, non-differential misclassification and underreporting of diagnoses may have attenuated the association between vitamin D status and COPD.The incidence of COPD is likely an underestimate of the true incidence since not all COPD patients are admitted to the hospital. Also, they may be admitted under different diagnoses, e.g. pneumonia. Finally, our study may not have enough power to show small-moderate effects of vitamin D on COPD risk, particularly in the fully adjusted models.

Regarding the validity of the methods used for the measurement of vitamin D status, it is recommended to store samples for measurement of 25-OH-D at −80°C, but several studies have shown 25-OH-D to be stable under various conditions [33]; [34]. Measuring serum 25-OH-D in general is associated with methodological issues, and variations between methods are considerable [35]. However, any misclassification is likely to be random and would have attenuated the associations towards the null value.

The difference in 25-OH-D concentrations between the studies could be due to several factors e.g. the different methods for measuring vitamin D, evaporation during storage, or a real decrease in vitamin D levels in the population over the years. Vitamin D status was used both as a continuous variable and in quartiles that was categorized before pooling of the studies. We adjusted for the different levels of vitamin D by adjusting for study population. There was no interaction between vitamin D status and study population. Results of analyses for each cohort separately were consistent with the combined analyses of the cohorts indicating that methodological differences between the cohorts did not seriously bias our results.

Baseline participation rates were relatively low. In general, non-participation is associated with less favourable socioeconomic characteristics [36], and Bender et al found strong socioeconomic inequality in participation at baseline in the Inter99 study [37]. Also, participants in population studies comparable to ours have found a higher age and a higher number of women among participants [14].Overall, this selection bias may limit the generalizability of the results to the general population, although studies with lower participation than ours were in fact not biased by nonparticipation [38].

Many questions regarding vitamin D status and (chronic obstructive pulmonary) disease still remain unanswered. Whether there is a continual or a threshold effect of vitamin D supplementation, whether to prefer bolus or steady state supplementation, and whether certain subgroups have larger effects than others are some of the unresolved issues. An extensive RCT (VITAL) funded by The National Institutes of Health and lead by Gold and Manson have started. It will follow 20,000 persons over 50 years as they are supplemented with 2,000 international units of vitamin D a day for five years. Another possibility is to use Mendelian randomization —a method of using genetic variants to examine the causal effect of a modifiable exposure on disease— to assess and quantify a possible causal effect of vitamin D on COPD [39].

In conclusion, we found a statistically significant inverse cross-sectional association between vitamin D status and COPD but found no association between vitamin D status and incident fatal and non-fatal COPD. Further studies, RCTs and genetic studies, will hopefully clarify the matter.
